# MMKP: A mind mapping knowledgebase prototyping tool for precision medicine

**DOI:** 10.3389/fimmu.2022.923528

**Published:** 2022-08-25

**Authors:** Siliang Liang, Yun Li, Qingling Dong, Xin Chen

**Affiliations:** Institute of Pharmaceutical Biotechnology and the First Affiliated Hospital Department of Radiation Oncology, Zhejiang University School of Medicine, Hangzhou, China

**Keywords:** precision medicine, knowledgebase, polymorphic foreign key, mind map, prototyping tool

## Abstract

**Background:**

With significant advancements in the area of precision medicine, the breadth and complexity of the relevant knowledge in the field has increased significantly. However, the difficulty associated with dynamic modelling and the disorganization of such knowledge hinders its rapid development potential.

**Results:**

To overcome the difficulty in using the relational database model for dynamic modelling, and to aid in the organization of precision medicine knowledge, we developed the Mind Mapping Knowledgebase Prototyping (MMKP) tool. The MMKP implements a novel design that we call a “polymorphic foreign key”, which allows the establishment of a logical linkage between a single table field and a record from any table. This design has advantages in supporting dynamic changes to the structural relationships in precision medicine knowledge. Knowledge stored in MMKP is presented as a mind map to facilitate human interaction. When using this tool, medical experts may curate the structure and content of the precision knowledge in a flow that is similar to the human thinking process.

**Conclusions:**

The design of polymorphic foreign keys natively supports knowledge modelling in the form of mind mapping, which avoids the hard-coding of medical logic into a rigid database schema and significantly reduces the workload that is required for adapting a relational data model to future changes to the medical logic. The MMKP tool provides a graphical user interface for both data management and knowledgebase prototyping. It supports the flexible customization of the data field constraints and annotations. MMKP is available as open-source code on GitHub: https://github.com/ZjuLiangsl/mmkp.

## Background

Precision medicine is a domain that was built on the border between medicine, genetics and information technology that led to the development of modern medical sciences and technologies (1). A core objective of precision medicine is to build a comprehensive knowledge network that illustrates the molecular mechanisms of individual diseases through integration and analysis of a large scale of samples and massive amounts of data (2). This allows prevention and treatment to be personalized according to an individual’s characteristics, such as their genomic traits (3). Through the years, precision medicine has guided numerous genetic traits as medical indicators into clinical practice. A series of evolving theories on the effective usages of these indicators are associated with these indicators (4–6). The modelling, organization and management of such precision medicine knowledge is a challenge because of its dynamic nature. Existing database solutions presently need to manage large amounts of data with nonvolatile relationships (7–9).

The state-of-the-art knowledgebase management solutions, including knowledgebases that manage precision medicine knowledge, mostly use the relational database management system (RDBMS) model. RDBMS supports recursive query and is able to query the transfer relationship in a general data model (10). While RDBMS is convenient for storing and querying a large amount of data with a stable relational structure, making changes to the data structure in an RDBMS is difficult. It was argued that RDBMS is incompetent for the management of fast-evolving precision medicine knowledge (11). Precision medicine knowledge is typically made of a large number of different categories of information, where the pieces of information in each category are not as abundant as compared to other domains of database applications. The structural complexity of the precision medicine knowledgebase built by RDBMS adds to the complexity when the relational structure of the database needs to be changed to reflect the addition of a new type of knowledge (12, 13). An example is shown in a later section to illustrate this deficiency.

More recently, researchers have also tried non-RDBMS technologies for building precision medicine knowledgebases. Examples include semantic networks (12), distributed file systems (13) and graph databases (14, 15). Semantic networks describe knowledge in the form of triplets, which have a limited expression power (16) and are difficult to use when modelling natural language-based precision medicine knowledge. The distributed file system and graph database, also known as the NoSQL database, are relatively new and still lack a medical expert-friendly integrated development environment and a globally-accepted standard (17). In this regard, RDBMS-based relational modelling and data management are still competitive approaches; it exhibits good semantic capturing abilities and technical stability. RDBMS is still the first choice when a new precision medicine knowledgebase is being developed.

This work presents MMKP, a fast-prototyping tool for precision medicine knowledgebase systems that uses RDBMS as a backend. MMKP supports the modelling of precision medicine knowledge with a new design pattern that we call a polymorphic foreign key, which significantly improves the capability of the resulting knowledgebase to adapt to changes in knowledge structures. This tool also supports the visualized curation of a knowledgebase schema and knowledge content in the form of a mind map, which is a human-friendly way to model and express medical knowledge (18, 19). In addition, users may alternatively revise knowledge structures and knowledge content, which simulates the flow of a human expert learning new knowledge.

## Results

### The schematic design of the MMKP

In the area of knowledge representation, scientists have discovered that a semantic unit may be abstracted as a category or an entity. A knowledge entity is an object or a phenomenon recognized by an individual, while a knowledge category is the cognition acquired by abstracting a class of entities of which the commonalities are shared. Traditionally, when implementing a relational data model, it is necessary to first go through the domain knowledge and extract all the categories (20). Database tables are then established with fields corresponding to these categories of knowledge. Foreign key relationships are established to constrain a table filed to be associated with the record of another table. This procedure adapts well to the domains of knowledge modelling where the knowledge structure remains stable. However, in the area of precision medicine, new types of principles, theories or mechanisms are frequently proposed and integrated into practice (21, 22). Consequently, there is a frequent need to add new knowledge categories into the existing knowledgebases. It is virtually impossible for a knowledgebase designer to know *a priori* the complete list of categories that modelling certain precision knowledge would require. Instead, additional categories of knowledge must be continuously integrated with the existing system. Therefore, a tool that supports interactive knowledge structures and knowledge content development, i.e., a tool that supports alternative curation and revision of the knowledge structure and knowledge content, would be desirable. Such a tool will require a nontraditional design to enable its capability to model dynamic knowledge structures.

MMKP implements a new design to represent knowledge linkages instead of using the foreign key constraint. This design is called a polymorphic foreign key. A polymorphic foreign key can be intuitively understood as a pair of identifiers, one of which identifies a table, and the other of which identifies a record that can be connected in this tables. In contrast to the foreign key constraint, a polymorphic foreign key enables one table field to connect with a record that may be stored in multiple potential tables. This design enables efficient revision of the knowledge structure, where new types of knowledge (tables) may be easily wired into the existing knowledge system. For example, the practice of disease diagnosis may be modelled as a flow of action steps. Each step has a “next step”. However, it is difficult for us to know *a priori* all possible types of diagnostic actions (e.g., do some tests, prepare a room, etc.) and design their tables accordingly. We expect new types of diagnostics (new tables) to be integrated into the knowledgebase constantly. In MMKP, we create a field called the “next step” with its data type set to a polymorphic foreign key (which is denoted “reference” for short in the MMKP interface). This field may store an identifier to link the current step (the current record) to any type of next step (a record in another table). New types of diagnostic steps can therefore be easily integrated into the knowledge system by adding new tables, and existing knowledge can link with new knowledge through the polymorphic foreign key field. A more detailed discussion is given later in the section with an example.

### Knowledge modelling of a precision medicine guide using the polymorphic foreign key

The Clinical Pharmacogenetics Implementation Consortium (CPIC) is a source of well-established pharmacogenetic dosing guidelines. The organizations of precision medicine knowledge in CPIC are generalizable examples for evaluating whether a knowledge modelling tool can effectively process precision medicine knowledge. We illustrate the usage of MMKP modelling a point-of-care clinical decision support (CDS) in the CPIC Guideline for one of the thiopurines, 6-Mercaptopurine (6-MP), with the Thiopurine methyltransferase (TPMT) and *NUDT15* genotypes. Detailed information can be found in **Table 1**.

**Table 1:** Web locations of CPIC information.

Description	Location
CPIC Guideline for Thiopurines and TPMT and NUDT15	https://cpicpgx.org/guidelines/guideline-for-thiopurines-and-tpmt/
Most recent guideline publication	https://files.cpicpgx.org/data/guideline/publication/thiopurines/2018/30447069-supplement.pdf
TPMT consult and implementation workflow	https://files.cpicpgx.org/data/report/current/gene_cds/TPMT_CDS.xlsx
Mercaptopurine pre- and post-test alerts	https://files.cpicpgx.org/data/guideline/publication/thiopurines/mercaptopurine_Pre_and_Post_Test_Alerts_and_Flow_Chart.xlsx
Thioguanine pre- and post-test alerts	https://files.cpicpgx.org/data/guideline/publication/thiopurines/thioguanine_Pre_and_Post_Test_Alerts_and_Flow_Chart.xlsx

**Figure 1:**
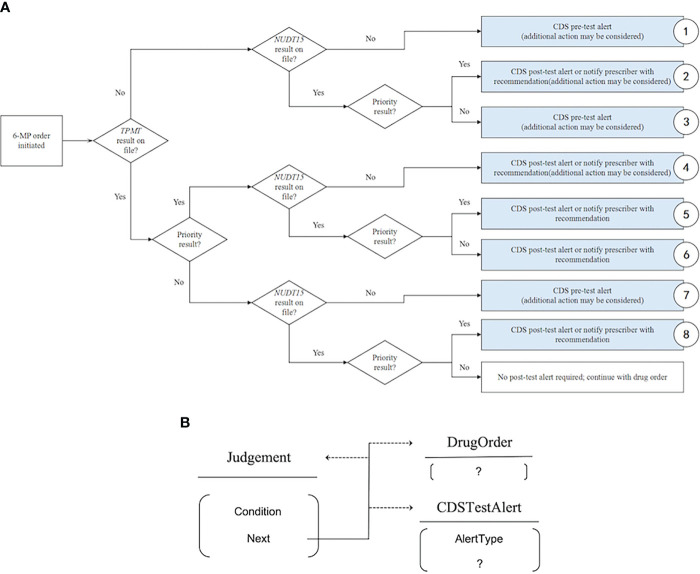
Decision flow in the CDS and its modelling. **(A)** The decision flow in dosing guidelines. **(B)** MMKP modelling of the decision flow using a polymorphic foreign key.

This CDS document presents precision medicine knowledge as a decision flow, which connects different diagnostic states to different next steps according to the decision criteria in the flow. Three categories of actions may be taken for a patient in a specific diagnostic state. These categories of actions include judgement, drug order suggestion and test alert suggestion. In the CDS document, judgement is represented by a rhomboid box with a criterion on a patient’s measurement. Drug order suggestions and test alert suggestions are represented by rectangular boxes with white and blue backgrounds, respectively (**Figure 1A**).

In MMKP modelling of the CDS, the judgement table contains a criterion based on which patients in a specific diagnostic state may be directed to the next step. The next step may be a judgement, a drug order suggestion or a test alert suggestion. The linkages between the “next step” field of the judgement table and the actual next step description (stored as records in the judgement table, drug order suggestion table and test alert suggestion table) are implemented by polymorphic foreign keys (**Figure 1B**). With this approach, the entire CDS is therefore stored in three tables by the MMKP. As in this example, it is expected that more types of actions that are defined by different specifications and require the storing of different tables need to be integrated into the current CDS when knowledge advances. Because the design of polymorphic foreign keys allows the dynamic addition of new types of table records into the possibilities that a field may link to, the addition of new types of actions is natively supported by an MMKP knowledgebase, which, in contrast, would require a complex effort in a traditionally designed knowledgebase. A hypothetical example is given later.

### The MMKP tool

**Figure 2:**
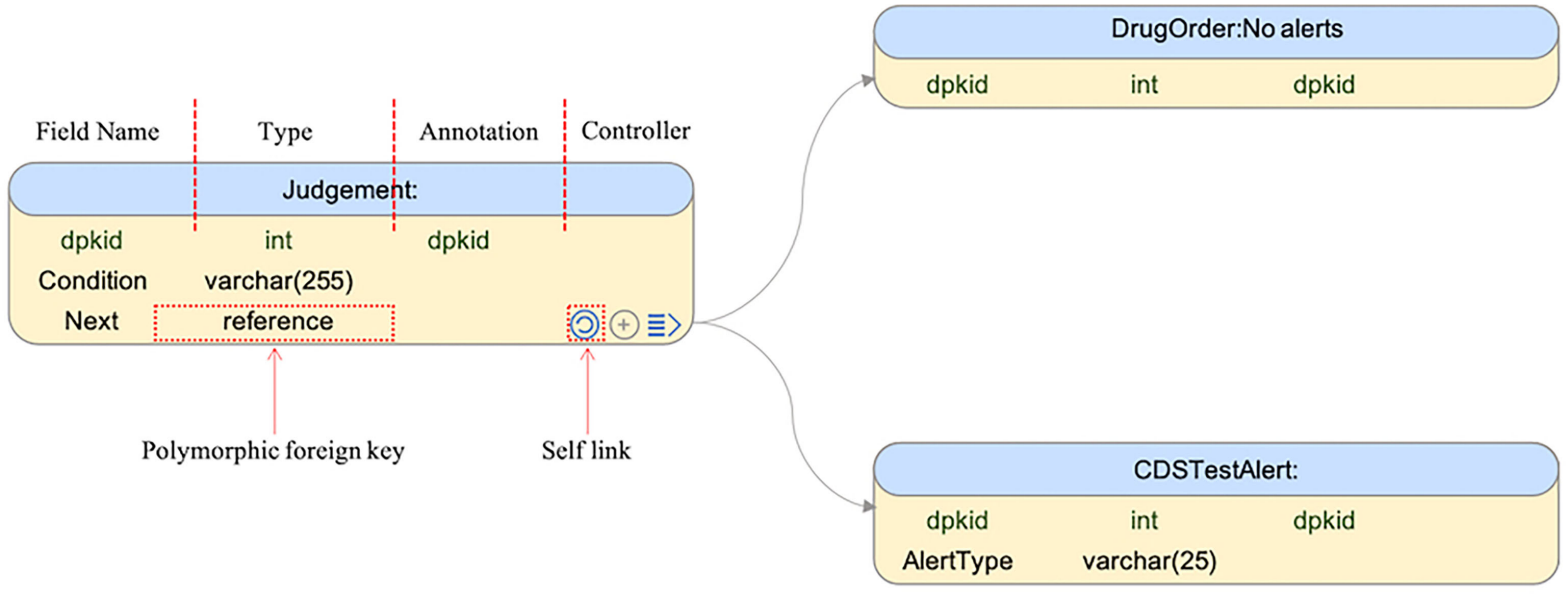
Interface of the MMKP schema design. Three knowledge tables abstracted from the decision flow are shown, of which the cells from left to right refer to the field name, field type, manual annotation and polymorphic foreign key controller. The Type ‘Reference’ indicates that field ‘Next’ is a polymorphic foreign key. The Controller button from left to right adds the polymorphic foreign key link to its main table, to a new table, or shows the already-linked tables in the working canvas.

The current version of the MMKP tool provides a mind mapping interface for designing table structures and the linkages between tables. An example showing the process of modelling 6-MP usage within patients with the *TPMT* and *NUDT15* genotypes, the CDS, is illustrated in **Figure 2**. In this example, the table “Judgement” is logically linked to the “Judgement” table itself, to the “DrugOrder” table and to the “CDSTestAlert” table through the field “Next” with the data type “polymorphic foreign key”.

**Figure 3:**
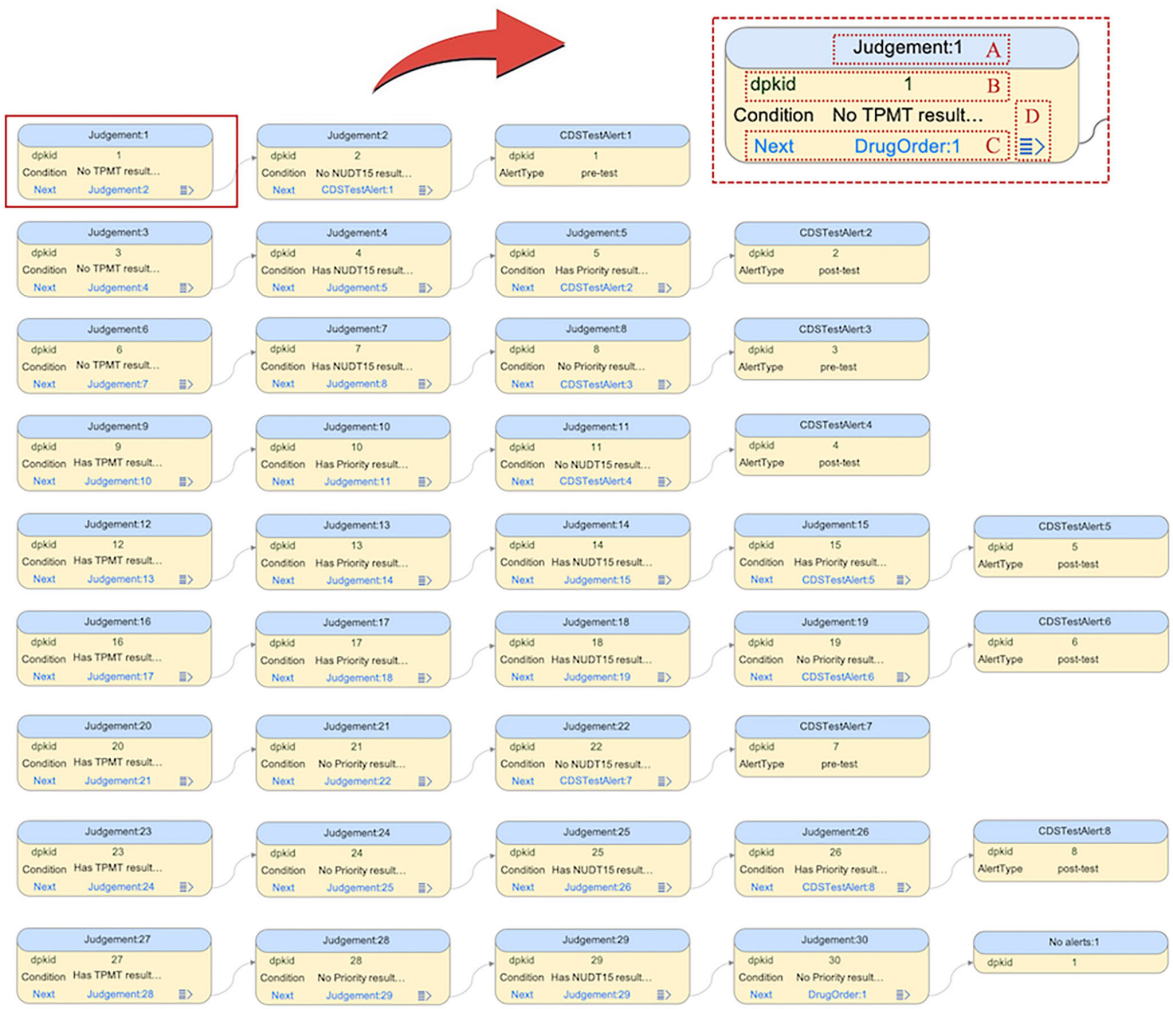
Interface of MMKP knowledge entry. Each cell frame represents a judgement or a decision in the decision flow. **(A)** Each frame is identified by its table name and primary key, which is quoted by the polymorphic foreign key. **(B)** Only tables with a single unique key can be linked by polymorphic foreign keys. The MMKP tool will automatically create a unique self-incrementing key for each table record, and this key is marked in green. **(C)** The value of the polymorphic foreign key is definite and unique and is marked blue. Users can drag and drop to make connections between a polymorphic foreign key field and a table record. **(D)** Users can choose to display or hide the downstream tables as necessary by expanding the controller.

After the knowledge structure is modelled, the MMKP provides an interface for knowledge entry. As in a typical clinical decision-making flow, each decision leads to a “next step”. When entering one step (table record), the user is allowed to leave the “next step” field blank at first. After the next step description has been recorded in its own table, the user can link this record back to the “next step” field of the record representing its previous step. All records are shown on a graphical canvas, and the linkages can be created and modified by drag and drop. This design facilitates knowledge entry by intuitively showing the logical relationship between logically connected records that spread over multiple tables (**Figure 3**). The MMKP tool ensures the constraint that a polymorphic foreign key field links only to one record in a table. This method of knowledge entry simulates the method of human thinking and is therefore more user-friendly than existing database management tools that rely on the traditional relation data model.

In addition to knowledge modelling, the current version of the MMKP tool supports a range of auxiliary functions, such as metadata management and data dictionaries, as well as a flexible tool to specify table field constraints. The MMKP tool is implemented with Java. It uses an open source Vue-based mind map module as an interface and uses the Springboot framework for project management.

Internally, the MMKP tool uses a meta-database and an entity base for data organization and data buffer management. This design ensures the stability of the data source by making it relatively isolated from user activities. Knowledge structures and knowledge entries created by MMKP may be exported and stored in a variety of backend database management systems for subsequent development. The source code for MMKP is available at https://github.com/ZjuLiangsl/mmkp.

## Discussion

### A polymorphic foreign key is an efficient design for modelling dynamic knowledge

In the above example, knowledge modelling by the MMKP is achieved by three tables, corresponding to the three types of knowledge that logically require their own table to store. The logical links between these tables are stored in a polymorphic foreign key field, which avoids stereotyping known medical logic into a materialized database schema and allows for future extension. In the MMKP, the polymorphic foreign key field actually models a higher-level concept of the human mind (the “next step” concept in this example). The MMKP approach may therefore be regarded as a higher-level abstraction of a knowledge structure. This higher-level abstraction can remain stable when a lower-level abstraction (data fields that make up a table) changes. This pattern of higher-level abstraction is frequently found in natural human thinking. MMKP is a tool that is capable of such higher-level modelling of knowledge.

**Figure 4:**
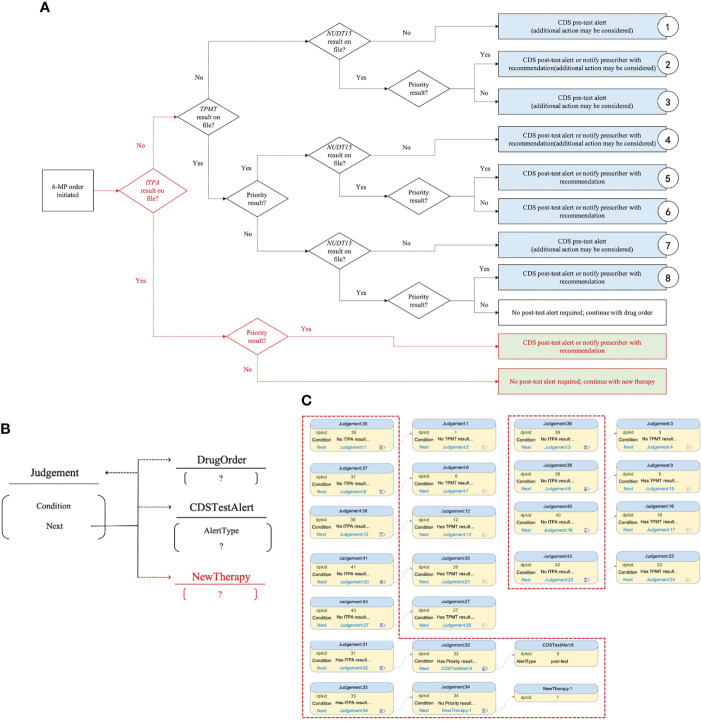
Change of decision flow modelled by MMKP. **(A)** Addition of a new genotype test result *ITPA* and its new therapy for patients. **(B)** Knowledge content reflecting the new decision flow in MMKP. **(C)** Knowledge content entry in MMKP, with the updated content in a red dashed box.

This higher-level abstraction of knowledge bestows the advantage of a more stable knowledgebase schema, with new types of knowledge (data tables) easily being incorporated into the existing system. Below, we show an example. Assume that new evidence emerges that new genotype test result of inosine triphosphate pyrophosphatase (ITPA) and its new therapy have become available for patients. Subsequently, the decision flow needs to be modified as shown in **Figure 4**. In the MMKP, the necessary changes include, first, adding a new table to store the therapy specification and then rewiring the polymorphic foreign key links accordingly. There is no modification of the existing table structure or table constraint needed. This process is intuitive and safe and alleviates the subsequent need to modify knowledgebase application codes to accommodate the new change.

**Figure 5:**
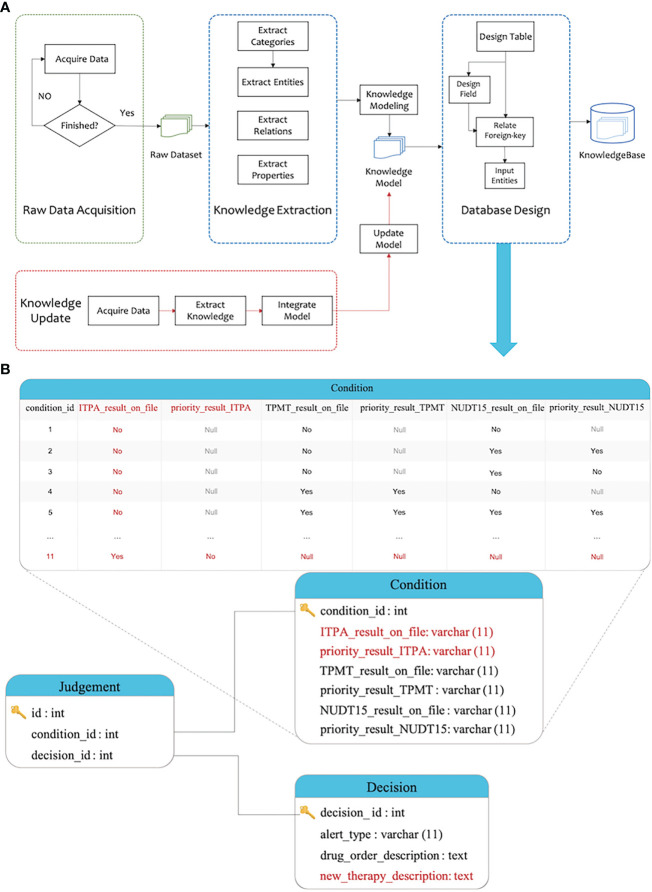
Modelling of CDS and its change with the traditional approach. **(A)** The traditional approach requires complex modifications to accommodate a new data table. **(B)** The knowledge content in the traditionally modelled knowledgebase. Table records need to be modified when a new data table is to be integrated into the knowledgebase.

In contrast, the traditional way of designing a relational database follows the database schema design norms (17). A typical design is shown in **Figure 5**, which includes three tables: “Condition”, “Decision”, and “Judgement”. The “Condition” table stores a patient’s measurements for decision-making, which are stored in different fields that correspond to whether the patient has “TPMT_result_on_file”, “NUDT15_result_on_file”, etc. The “Decision” table stores a collection of outcomes of the decision-making flow, which includes the fields of “alert_type” and “drug_order_description”. The “Judgement” table stores the correspondence between the condition table and the decision table. To accommodate the change that new genotype test result and therapy for patients becomes available, a number of changes, including data structure changes, are needed. New fields ‘ITPA_result_on_file’ and ‘priority_result_ITPA’ must be added into table “Condition”, which leads to the necessary action to modify all existing records in the condition table. In addition, a detailed description of the new therapy needs to be recorded. A new field ‘new_therapy_description’ needs to be added to table “Decision”, which also requires modification of all records in this table. Compared to the MMKP process, implementing this process requires a careful plan, is nonintuitive, and leads to extensive downstream work updating the database access codes for the knowledgebase application.

Outside the domain of database management, the action of linking one object to multiple objects of different types is also needed. For example, when building a web application, one content item is frequently needed to be linked to multiple other content items of different types. The Django web framework provides a Java based programming model to implement such links from one instance of a specific content type class to multiple instances of other content type classes. By this means, the Django web framework supports the development of reusable web applications, web sites and web tools. In the Django web framework, such technology is called “generic foreign keys”. Comparing to the generic foreign keys technology, our polymorphic foreign key technology provides a database management system-based solution to the need of complex links, which manages information instead of web components, and provides a method for data curators to model higher-level abstract concepts in scientific information.

### Precision medicine knowledge requires a dynamic modelling tool

As discussed previously, the traditional database design norm requires medical researchers to fully collect all categories and entities in a knowledge domain to compose a schema. Because of the lack of flexibility of the foreign key constraint, connection from a high-level concept (e.g., “next step”) to its real meaning (a record in a table) typically requires multiple foreign key fields if this high-level concept can refer to things that belong to different knowledge categories. Each foreign key field links to one category. Later, an addition to the schema would be complex and error prone and requires substantial downstream work. This approach is not sufficient to meets the needs of precision medicine knowledge management that deals with highly complex data and rapidly evolving knowledge categories.

Moreover, it is difficult to train medical experts and computer experts with expertise from each other (23, 24). The traditional model of knowledgebase design requires well-trained computer experts who understand medical knowledge to create a database schema (25). Due to the complexity and dynamic nature of medical knowledge, this process is often of very low efficiency, prone to misunderstanding and lasts for multiple cycles. The MMKP tool facilitates this process by allowing medical experts to express their knowledge using high-level concepts, leading to a more stable schema, as well as allowing for incomplete collection of knowledge categories at the initial stage, and permitting integration of new knowledge categories into the existing system with low costs.

**Figure 6:**
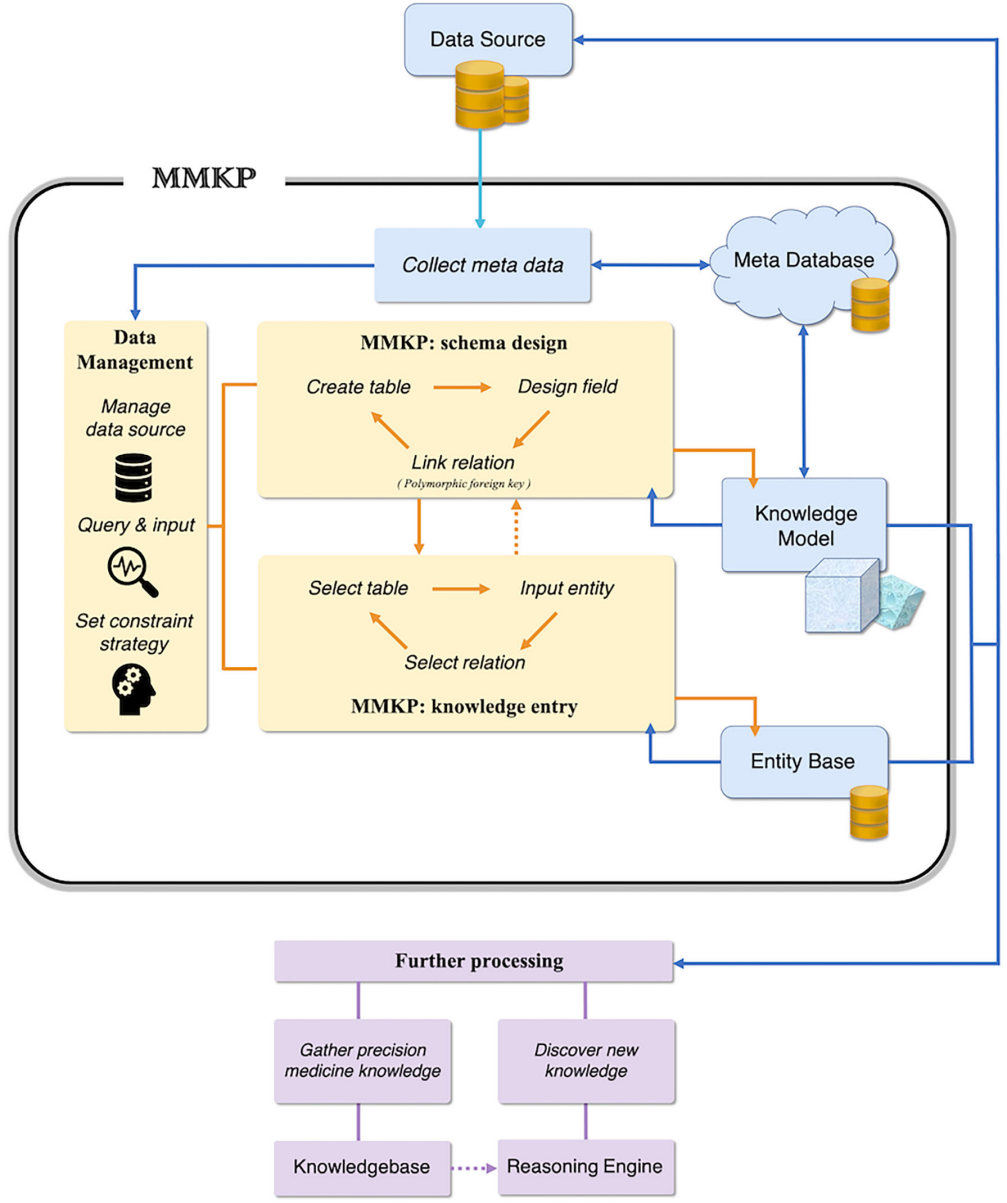
The schematic design of MMKP. MMKP applies a cyclic schema to model the precision knowledge and enter content, which supports alternative curation and revision of the knowledge structures and knowledge content. MMKP stores models and entities in the inner meta-database and entity base, keeping user operations independent of the data source before exporting.

With the efficient polymorphic foreign key design, the MMKP tool supports a data modelling process that simulates human thinking. MMKP allows alternative curation of the data schema and data content, i.e., users may create and modify knowledge structures and knowledge content in any order that is intuitive to the user. The curation of data content in the middle of a process curating a knowledge structure may also serve as a validation step to check whether the knowledge structure is correct and comprehensive (**Figure 6**).

With these features, MMKP demonstrates advantages over traditional database design tools such as ‘Sequel Pro’ (26) and ‘MySQL Workbench’ (27, 28) in modelling precision medicine knowledge, which are more suitable for precision medicine knowledgebase prototyping.

Currently, knowledge management for precision medicine faces multiple challenges including expert training, knowledgebase curation, and maintenance. MMKP provides a user-friendly mind mapping interface for domain experts to curate knowledgebases, which may facilitate the training of curation experts. When adopted widely, MMKP is expected to notably reduce the amount of time and effort that is necessary for domain expects to curate data. In addition, because that the data structure used by MMKP follows the thinking model of domain expects instead of programming specialists, knowledge stored in such structure is easy to understand and extend, which also facilitates the collaboration between different data curation teams to connect their knowledgebases. After a knowledgebase is initially created with MMKP, as illustrated before, extending it to include the latest discoveries is also simpler than knowledgebases created by other tools.

### Limitations and future of the MMKP

Precision medicine is a rapidly developing area, yet its translation to clinically proven patient benefits is still difficult. One of the bottlenecks is the information management efficiency. There are a greater number of precise indicators that may lead to different optimal decisions than those that a medical practitioner can remember and recall. However, the creation of a knowledgebase with the traditional data modelling approach is hindered by the fast-evolving knowledge structures in this field. The MMKP provides a solution for the efficient modelling and organization of precision medicine knowledge; however, this knowledge is still static. MMKP is not a tool that can help medical practitioners use this knowledge.

Medical artificial intelligence that supports clinical decision-making holds promise in breaking through this bottleneck. However, the development of such medical artificial intelligence is also hindered by the lack of a stable approach to represent medical knowledge, without which efficient reasoning algorithms are impossible to develop. In this regard, MMKP provides a way of representing precision medicine knowledge with higher-level concepts, leading to a more stable table schema. This advantage may also facilitate the development of efficient medical artificial intelligence applications on top of the MMKP data schema.

## Conclusions

In this paper, we introduce a precision medicine knowledgebase fast prototyping tool, MMKP. It uses a polymorphic foreign key to associate a higher-level concept with knowledge pieces from multiple knowledge categories, allowing alternative creation and modification of knowledge structures and knowledge content. It provides a web interface capable of knowledge modelling, knowledge content entry and exportation of curated knowledgebase prototypes to a series of database system backends. It provides a web interface that is graphical and was inspired by the form of a mind map. The MMKP tool is open source and available in GitHub.

## Data availability statement

The original contributions presented in the study are publicly available. This data can be found here: https://github.com/ZjuLiangsl/mmkp.

## Author contributions

SL and XC designed the MMKP. SL developed the MMKP. QD, YL and XC contributed valuable discussions in regard to the program design of the MMKP. SL and XC wrote substantial parts of the manuscript, and all authors proofread and approved the final version of the manuscript.

## Funding

This work was financially supported by the National Key Research and Development Program (2021YFC2100601), the National Natural Science Foundation of China (81830073).

## Acknowledgments

We thank Dr. Jin Jie for suggestions on website design.

## Conflict of interest

The authors declare that the research was conducted in the absence of any commercial or financial relationships that could be construed as a potential conflict of interest.

## Publisher’s note

All claims expressed in this article are solely those of the authors and do not necessarily represent those of their affiliated organizations, or those of the publisher, the editors and the reviewers. Any product that may be evaluated in this article, or claim that may be made by its manufacturer, is not guaranteed or endorsed by the publisher.

## Abbreviations

MMKP, Mind Mapping Knowledgebase Prototyping; RDBMS, relational database management system; CPIC, The Clinical Pharmacogenetics Implementation Consortium; CDS, clinical decision support; 6-MP, 6-Mercaptopurine; TPMT, Thiopurine methyltransferase; ITPA, inosine triphosphate pyrophosphatase.
